# Synaptic protein CSF levels relate to memory scores in individuals without dementia

**DOI:** 10.1186/s13195-025-01703-z

**Published:** 2025-03-03

**Authors:** Kirsten E. J. Wesenhagen, Diederick M. de Leeuw, Jori Tomassen, Johan Gobom, Isabelle Bos, Stephanie J. B. Vos, Pablo Martinez-Lage, Mikel Tainta, Julius Popp, Gwendoline Peyratout, Magda Tsolaki, Rik Vandenberghe, Yvonne Freund-Levi, Frans Verhey, Simon Lovestone, Johannes Streffer, Valerija Dobricic, Kaj Blennow, Philip Scheltens, August B. Smit, Lars Bertram, Charlotte E. Teunissen, Henrik Zetterberg, Betty M. Tijms, Pieter Jelle Visser, the Alzheimer’s Disease Neuroimaging Initiative

**Affiliations:** 1 Department of Neurology, Alzheimer Center Amsterdam, Amsterdam Neuroscience, Vrije Universiteit Amsterdam, Amsterdam UMC, VUmc, De Boelelaan 1118, 1081 HZ Amsterdam, the Netherlands; 2 Clinical Neurochemistry Lab, Institute of Neuroscience and Physiology, Sahlgrenska University Hospital, Mölndal, Sweden; 3 Institute of Neuroscience and Physiology, Department of Psychiatry and Neurochemistry, University of Gothenburg, Mölndal, Sweden; 4 Nivel, the Netherlands; 5 Department of Psychiatry and Neuropsychology, School for Mental Health and Neuroscience, Alzheimer Center Limburg, Maastricht University, Maastricht, the Netherlands; 6 Center for Research and Advanced Therapies, CITA-Alzheimer Foundation, Donostia-San Sebastian, Spain; 7 CITA-Alzheimer Foundation, San Sebastian, Spain; 8 Geriatric Psychiatry, Department of Mental Health and Psychiatry, Geneva University Hospitals, Geneva, Switzerland; 9 Department of Psychiatry, University Hospital of Lausanne, Lausanne, Switzerland; 10 Department of Neurology, Medical School, Faculty of Health Sciences, AHEPA University Hospital, Aristotle University of Thessaloniki, Thessaloniki, Makedonia Greece; 11 Neurology Service, University Hospitals Leuven, Louvain, Belgium; 12 Laboratory for Cognitive Neurology, Department of Neurosciences, KU Leuven, Louvain, Belgium; 13 Department of Neurobiology, Care Sciences and Society, Division of Neurogeriatrics, Karolinska Institutet, Stockholm, Sweden; 14 School of Medical Sciences öRebro University and Department of Psychiatry, öRebro University Hospital, Örebro, Sweden; 15 Alzheimer Center Limburg, School for Mental Health and Neuroscience, Maastricht University, Maastricht, the Netherlands; 16 Janssen-Cilag, Buckinghamshire, UK; 17 University of Oxford, Oxford, UK; 18 AC Immune SA, LLC. Beerse, Formerly Janssen R&D, Lausanne, Switzerland; 19 Department of Biomedical Sciences, University of Antwerp, Antwerp, Belgium; 20 Lübeck Interdisciplinary Platform for Genome Analytics (LIGA), University of Lübeck, Lübeck, Germany; 21 Clinical Neurochemistry Lab, Sahlgrenska University Hospital, Mölndal, Sweden; 22 Department of Molecular and Cellular Neurobiology, Center for Neurogenomics and Cognitive Research, Amsterdam Neuroscience, VU University Amsterdam, Amsterdam, The Netherlands; 23 Center for Lifespan Changes in Brain and Cognition (LCBC), Department of Psychology, University of Oslo, Oslo, Norway; 24 Neurochemistry Lab, Department of Clinical Chemistry, Amsterdam Neuroscience, Vrije Universiteit, Amsterdam, the Netherlands; 25 Department of Neurodegenerative Disease, UCL Institute of Neurology, London, UK; 26 UK Dementia Research Institute, London, UK; 27 Kong Center for Neurodegenerative Diseases, Hong Kong, China

**Keywords:** Synaptic proteins, Cerebrospinal fluid proteomics, Memory performance, Early Alzheimer’s disease

## Abstract

**Background:**

We investigated how cerebrospinal fluid levels of synaptic proteins associate with memory function in normal cognition (CN) and mild cognitive impairment (MCI), and investigated the effect of amyloid positivity on these associations.

**Methods:**

We included 242 CN (105(43%) abnormal amyloid), and 278 MCI individuals (183(66%) abnormal amyloid) from the European Medical Information Framework for Alzheimer's Disease Multimodal Biomarker Discovery (EMIF-AD MBD) and the Alzheimer’s Disease Neuroimaging Initiative (ADNI). For 181 (EMIF-AD MBD) and 36 (ADNI) proteins with a synaptic annotation in SynGO, associations with word learning recall were analysed with linear models.

**Results:**

Subsets of synaptic proteins showed lower levels with worse recall in preclinical AD (EMIF-AD MBD: 7, ADNI: 5 proteins, none overlapping), prodromal AD (EMIF-AD MBD only, 27 proteins) and non-AD MCI (EMIF-AD MBD: 1, ADNI: 7 proteins). The majority of these associations were specific to these clinical groups.

**Conclusions:**

Synaptic disturbance-related memory impairment occurred very early in AD, indicating it may be relevant to develop therapies targeting the synapse early in the disease.

**Supplementary Information:**

The online version contains supplementary material available at 10.1186/s13195-025-01703-z.

## Background

Memory impairment is a key feature of Alzheimer’s disease (AD) [13, 14]. Decline in memory function precedes dementia onset, starting already in the preclinical stage of AD [15]. A better understanding of mechanisms underlying memory loss can help the development of novel therapies.

Previous studies have shown that memory scores are associated with reduced synaptic density [27, 28, 35–40] but this research was mostly conducted post-mortem in AD patients who were in late disease stages. Synaptic protein levels can also be assessed in vivo in cerebrospinal fluid (CSF). So far, studies have focused on few or single synaptic markers in CSF. For example, neurogranin (NRGN) is a widely studied postsynaptic protein, showing higher levels with worse memory performance in prodromal AD [12], although contradicting results have been found for memory associations in cognitively normal (CN) individuals [9, 12, 19] and in AD dementia [29, 41]. Associations of synaptosomal-associated protein 25 (SNAP25) and neuromodulin (also called growth-associated protein 43, GAP43) with memory functioning depended on disease stage, with associations in early but not in later AD [31]. Many additional synaptic proteins exist, for which it is largely unknown if they correlate with memory functioning in the early stages of AD before dementia onset. Furthermore, individuals can have mild cognitive impairment (MCI) with *normal* amyloid (i.e., non-AD MCI), and it remains unclear what mechanisms may explain impaired memory in such individuals.

Using a proteomics approach of CSF, we aimed to analyse which synaptic proteins are related to memory scores in older non-demented individuals. We hypothesized that synaptic protein levels in CSF would be associated with worse memory scores, and that a subset of these associations would be specific for AD pathology and/or depend on cognitive stage. In two independent cohorts, we analysed cross-sectional associations of synaptic protein levels in CSF with memory scores in preclinical AD and prodromal AD, and in CN amyloid normal controls and non-AD MCI, to test if memory associations were specific for AD pathology.

## Methods

### Study participants

Two independent study cohorts, the European Medical Information Network Alzheimer’s disease multi-modal biomarker discovery study (EMIF-AD MBD) [5] and Alzheimer’s Disease Neuroimaging Initiative (ADNI) [1] were used in these analyses. All participants provided informed consent to participate in these studies. We selected individuals who were CN and individuals with a clinical diagnosis of MCI if they had memory test scores and CSF proteomic data available. In EMIF-AD MBD, CN was defined in all centres based on neuropsychological examination scores within 1.5 standard deviation (SD) of age-, sex- and education adjusted standards, and in four centres with additional criteria (see [5] for more details). MCI was defined using cohort-specific criteria (in all cohorts Petersen criteria [32] except for Lausanne, where Winblad criteria [48] were used and Clinical Dementia Rating (CDR) of 0.5).

The ADNI (adni.loni.usc.edu) was launched in 2003 as a public–private partnership, led by Principal Investigator Michael W. Weiner, MD. The primary goal of ADNI has been to test whether serial magnetic resonance imaging (MRI), positron emission tomography (PET), other biological markers, and clinical and neuropsychological assessment can be combined to measure the progression of mild cognitive impairment (MCI) and early Alzheimer’s disease (AD). For up-to-date information, see www.adni-info.org. Detailed information about the definitions of CN and MCI in ADNI, and methodology of the memory testing can be found in the general procedures manual of ADNI [1]. In short, CN was defined as absence of memory complaints, normal scores on the Logical Memory II subscale (delayed Paragraph Recall) from the Wechsler Memory Scaled – Revised, Mini-Mental State Examination (MMSE) score ≥ 24 and CDR of 0. MCI was defined by a memory complaint, abnormal scores on the Logical Memory II subscale, MMSE ≥ 24 and CDR of 0.5.

For the analyses, we characterized participants from EMIF-AD MBD and ADNI into four diagnostic groups depending on their clinical and amyloid status: CN individuals with normal amyloid (controls), CN individuals with abnormal amyloid (preclinical AD), MCI individuals with normal amyloid (non-AD MCI) and MCI individuals with abnormal amyloid (prodromal AD).

### CSF amyloid, t-tau and p-tau biomarkers

For EMIF-AD MBD, we used cut-points for amyloid status that were previously determined with gaussian mixture modelling for each center [44]. T-tau and p-tau were measured in local laboratories using either Innotest (Fujirebio Europe, Gent, Belgium) (4 cohorts), or with INNO-BIA AlzBio3 (Fujirebio Europe), and we used local cut-offs to determine abnormality [5]. In ADNI, CSF amyloid, t-tau and p-tau were measured using multiplex xMAP Luminex platform (Luminex Corp, Austin, TX) with INNO-BIA AlzBio3 (Fujirebio Europe), with abnormality defined as amyloid < 192 pg/ml, t-tau > 93 pg/ml and p-tau > 23 pg/ml.

### Proteomics

In EMIF-AD MBD, proteomics was performed with the tandem mass tag (TMT) technique using 10 + 1 plexing [5] as previously described [3, 25, 42]. In addition, in a central laboratory (Gothenburg University, Sweden), levels of neurofilament light (NEFL) were determined with the NF-Light assay (UmanDiagnostics, Umeå, Sweden) and NRGN with an in-house immunoassay [34]. All protein levels were natural log-transformed. This dataset included 2537 proteins in 306 individuals (missing values; mean 6%, range 0 to 99%).

In ADNI, two proteomic datasets were used. In the first dataset, proteomic data were generated using LC/MS-MRM (panel developed by Caprion Proteome Inc.)(“MRM Assays: Caprion,” n.d.) and the Rules-Based Medicine (RBM)(“Myriad RBM Operational Procedures White Paper—Myriad RBM,” n.d.) platform. These included 202 proteins in 214 individuals (missing values; mean 0.3%, range 0 to 45%). We used the pre-processed and quality checked normalized data publicly available on the ADNI website (adni.loni.usc.edu). We natural log-transformed and Z-scored individual RBM measurements and MRM peptide measurements. Next, we generated protein scores by averaging MRM peptides and correlating RBM proteins that were mapped to the same protein (r at least 0.5). Protein levels in both datasets were standardized relative to the mean and standard deviations from controls with normal amyloid, t-tau and p-tau.

Recently, another mass spectrometry proteomics analysis was performed in ADNI (CSF 48 panel) and provided data on 48 proteins in 706 individuals [11]. Proteomic data from this panel was used as the second dataset in this study. Our main analyses, however, were performed in the Caprion/RBM dataset as these included the largest number of proteins and the second dataset served for validation purposes.

### Synapse proteins

**Fig. 1:**
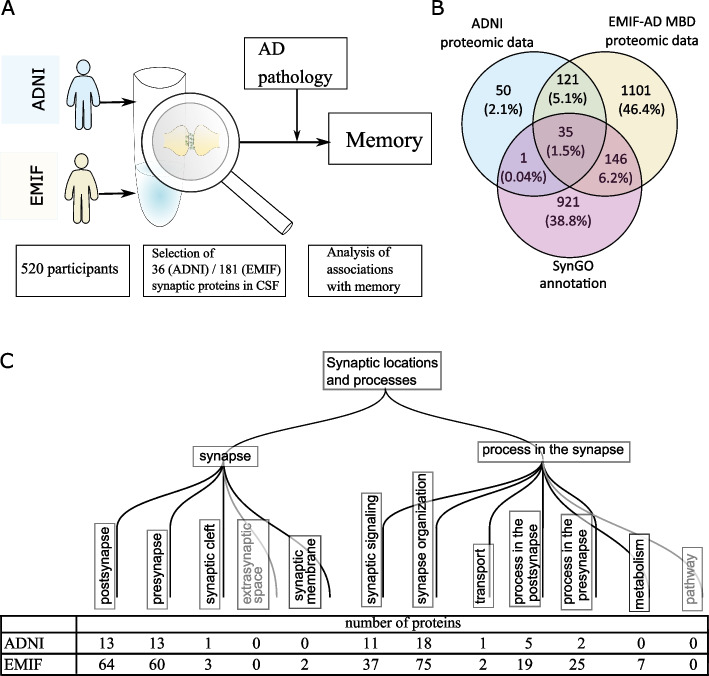
Characterization of synaptic proteomic dataset. **A** Schematic overview of study design. **B** Overlap of ADNI proteomics and EMIF-AD MBD proteomics with SynGO annotation used to select synaptic proteins. **C** SynGO locations and processes for which synaptic proteins were included in the datasets. One protein can have multiple annotations for different locations and processes. The SynGO annotation uses a hierarchical organisation, and we show here the first and second level of subprocesses, with proteins in lower subprocesses categorized with the respective second level subprocess

We used Synaptic Gene Ontologies to identify synaptic proteins (SynGO) [18],“SynGO—Synaptic Gene Ontologies and annotations,” n.d.), a curated database with detailed annotation of known synapse-related proteins. An additional criterion for inclusion of proteins in our analyses was that proteins were measured in at least 25 individuals per diagnostic subgroup. In ADNI Caprion/RBM and CSF 48 panel, respectively 36 synaptic proteins (17% of 202 total) and 10 synaptic proteins (21% of 48 total) were identified. In EMIF-AD MBD 181 synaptic proteins (13% out of 1350 total) were identified (Fig. 1).

### Memory tests

We measured memory functioning with both immediate and delayed recall memory tests, because immediate and delayed recall may differ in their sensitivity to detect decline in different stages of AD [4, 16, 45]. In EMIF-AD MBD, memory function was assessed with cohort specific tests (auditory verbal learning test (AVLT): 3 cohorts; Consortium to Establish a Registry for Alzheimer’s Disease wordlist (CERAD), Free and Cued Selective Reminding Test(FCSRT) and RI-48 in the remaining 3 cohorts). Test scores were Z transformed according to age, sex and education norms (as detailed in [5]), and values ranged between −5 to 5.

In ADNI, we used the Rey auditory verbal learning test (RAVLT, immediate recall: sum of correctly recalled words over 5 trials, range 0–75; delayed recall: correctly recalled words after 30-min delay, range 0–15). We transformed the memory scores into age-, education- and sex-adjusted Z-scores, using adjustment factors for age, education and sex that were estimated from linear models for memory performance of all ADNI controls.

### Statistical analyses

Differences in demographic variables between groups were assessed with chi-square test, Mann–Whitney U test, Student's t-test or Kruskal–Wallis test when appropriate. Unless indicated otherwise, effects were considered significant at a *p*-value < 0.05. We first tested if protein levels differed depending on diagnostic group with Analysis of variance (ANOVA), and if so, performed two-sided t-tests between the diagnostic groups. To account for the multiple comparisons (in total 6 tests), we considered differences between the diagnostic groups significant at a false discovery rate (FDR)-adjusted *p*-value < 0.05. Next, we tested associations between memory test scores (outcome) and synaptic protein level (predictor) with linear models that included the covariates diagnostic group, age, sex and years of education (model 1) and additionally the interaction between protein level and diagnostic group (model 2). To report the effects of protein levels on memory scores across diagnostic groups, we selected proteins with a diagnostic group-interaction *p*-value ≥ 0.1. To report the effects of protein levels on memory scores that depended on diagnostic group, we selected proteins if they had a significant interaction with diagnostic group (interaction *p*-value < 0.1), and calculated associations with memory scores separately in the different diagnostic groups. For reference, we also provide statistical details of both models for all proteins (Supplementary Table 1). We then tested if the memory-associated proteins in our analyses were enriched for specific synaptic functions with the SynGO website(“SynGO—Synaptic Gene Ontologies and annotations,” n.d.), and selected biological process ontology terms for the enrichment analyses when they included at least 3 genes. For visualisation purposes, we calculated composite scores of synaptic proteins, by averaging proteins which showed an interaction (interaction *p*-value < 0.1) with diagnostic group on immediate or delayed recall scores. All analyses were run in R 4.1.2 “Bird Hippie”. *Emmeans* 1.4.2 was used for estimation of regression coefficients.

## Results

We included 137 controls, 105 individuals with preclinical AD, 183 individuals with prodromal AD and 95 individuals with non-AD MCI (Table 1). In both studies, the preclinical and prodromal AD groups had more Apolipoprotein E (*APOE*) ε4 carriers and tended to have higher CSF t-tau and p-tau levels than controls. Compared to EMIF-AD MBD participants, ADNI participants were older (all groups), had received longer education (all groups) and had lower memory test scores (controls, preclinical and prodromal AD) (Table 1, Supplementary Fig. 1).

**Table 1:** Demographics of study participants in EMIF-AD MBD and ADNI

	EMIF-AD MBD				ADNI			
	Controls	Preclinical AD	MCI with normal amyloid	Prodromal AD	Controls	Preclinical AD	MCI with normal amyloid	Prodromal AD
n	87	73	66	80	50	32	29	103
Age in years, mean ± sd	65.9 ± 7.8^ad^	66.8 ± 8.3^af^	67.2 ± 8^ag^	70.3 ± 6.7^adfg^	75.3 ± 5.5^a^	76.1 ± 5.6^a^	75.3 ± 7.8^a^	74.7 ± 7.2^a^
Sex, female (%)	43(49%)	39(53%)	29(44%)^a^	45(56%)^a^	25(50%)^c^	15(47%)^e^	5(17%)^ace^	38(37%)^a^
APOE-e4 carriership, 1–2 alleles (%)	22(25%)^bd^	44(60%)^be^	21(32%)^aeg^	55(69%)^dg^	5(10%)^bd^	16(50%)^be^	3(10%)^aeg^	67(65%)^dg^
MMSE score, mean ± sd	28.9 ± 1.2^ cd^	28.7 ± 1.2^aef^	26.6 ± 2.6^ce^	26.7 ± 2.5^df^	28.9 ± 1^ cd^	29.2 ± 1^aef^	27.4 ± 1.8^ce^	26.8 ± 1.7^df^
Delayed recall Z-score, mean ± sd	0.444 ± 0.99^acd^	0.337 ± 0.95^aef^	−1.28 ± 1.1^ce^	−1.23 ± 1.2^adf^	0.0053 ± 1^acd^	−0.254 ± 1.2^aef^	−1.52 ± 1^ceg^	−1.99 ± 1.1^adfg^
Immediate recall Z-score, mean ± sd	0.523 ± 0.93^acd^	0.252 ± 0.97ef	−1.33 ± 1.4^ce^	−0.952 ± 1.3^adf^	0.128 ± 0.96^acd^	0.158 ± 1.1^ef^	−0.985 ± 1.1^ce^	−1.43 ± 1.2^adf^
Education in years, mean ± sd	12.7 ± 3.4^acd^	12.6 ± 3.6^aef^	10.5 ± 3.6^ace^	11.1 ± 3.4^adf^	15.6 ± 2.8^a^	15.7 ± 3.4^a^	16.4 ± 2.8^a^	15.8 ± 3^a^
CSF amyloid, mean ± sd	0 ± 1^bcd^	−1.42 ± 0.57^abe^	0.425 ± 1.2^ceg^	−1.26 ± 0.86^adg^	0 ± 1^bd^	−4.35 ± 1.6a^bef^	−0.0274 ± 0.89^eg^	−5.1 ± 1.4^adfg^
CSF t-tau, mean ± sd	0 ± 1^ cd^	0.726 ± 2.5^f^	0.791 ± 2.2^cg^	2.39 ± 2.2^dfg^	0 ± 1^bd^	1.06 ± 1.6^bef^	0.0525 ± 1^eg^	2.55 ± 2.6^dfg^
CSF p-tau, mean ± sd	0 ± 1^ cd^	0.242 ± 1.5^af^	0.318 ± 1.4^acg^	1.76 ± 1.7^dfg^	0 ± 1^bd^	1.01 ± 1.6a^bef^	−0.194 ± 0.69^aeg^	1.91 ± 1.6^dfg^

Memory scores in EMIF are age-, sex- and education-adjusted Z-scores based on published test reference values or local norms; memory scores in ADNI were age-, sex- and education-adjusted Z-scores based on memory scores within the ADNI cohort, using all available memory scores in this population for CN individuals. Levels of amyloid, t-tau and p-tau are Z-scores relative to the CN A- group

*CN* cognitively normal, *MCI* mild cognitive impairment

a-g, *p*-value < 0.05 for a: comparing same cognitive and amyloid group between EMIF-AD MBD and ADNI, b: comparing CN A + and CN A- in same dataset, c: comparing MCI A- and CN A- in same dataset, d: comparing MCI A + and CN A- in same dataset, e: comparing MCI A- and CN A + in same dataset, f: comparing MCI A + and CN A + in same dataset, g: comparing MCI A + and MCI A- in same dataset

**Fig. 2:**
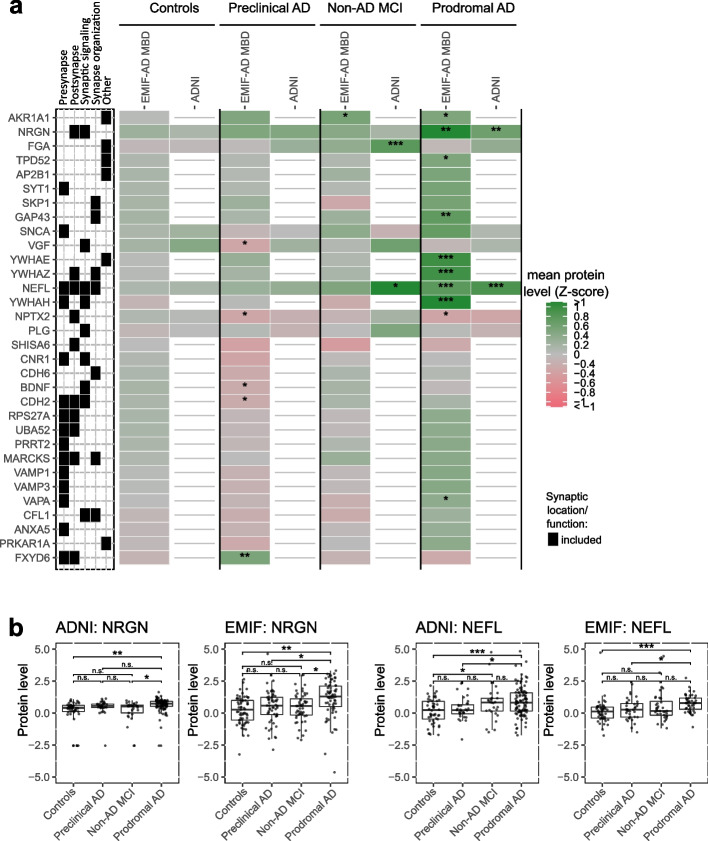
Differences in synaptic proteins depending on diagnostic group. **a** Mean protein levels are shown for top proteins depending on amyloid and cognitive subgroups, **b** boxplots of protein levels between diagnostic groups for neurogranin (NRGN) and neurofilament light (NEFL). The box of the boxplot indicates 25th percentile, median and 75th percentile, whiskers indicate 1.5 × interquartile range. See Supplementary Table 2 for mean differences between groups and full synaptic annotation for all proteins. Synaptic category ‘Other’ refers to other synaptic locations and functions as detailed in Fig. 1c. *, ** and *** indicate significant difference between diagnostic group and controls (**a**), or between indicated diagnostic groups (**b**): *, *p*-value < 0.05, **, *p*-value < 0.01; ***, *p*-value < 0.001, n.s.: not significant

We selected 181 synaptic proteins in EMIF-AD MBD and 36 synaptic proteins in ADNI that were present in at least 25 individuals for each diagnostic group (Fig. 1). In EMIF-AD MBD, 30 of these proteins differed between diagnostic groups (ANOVA *p*-value < 0.05; supplementary Table 2). Relative to controls, individuals with preclinical AD showed lower levels of VGF, NPTX2, BDNF, CDH2 and higher levels of FXYD6. Compared to controls, individuals with prodromal AD showed lower levels of NPTX2 and higher levels of 9 proteins (YWHAE, YWHAH, YWHAZ, NEFL, NRGN, GAP43, TPD52, VAPA and AKR1A1). The levels of these proteins were typically also higher in prodromal AD compared to preclinical AD and non-AD MCI (Fig. 2A, Supplementary Table 2, Supplementary Table 3). There was no specific association with synaptic locations (pre- and post-synapse) or synaptic functions (synaptic signalling or organization). In ADNI Caprion/RBM, only four proteins showed different levels between diagnostic subgroups (Fig. 2A, Supplementary Table 2). As in EMIF-AD MBD, NRGN and NEFL were increased in prodromal AD compared to controls, and were also increased compared to individuals with preclinical AD or non-AD MCI (Fig. 2B). FGA and PLG were increased in non-AD MCI relative to prodromal AD and/or controls.

In ADNI Caprion/RBM, we found that several proteins showed an opposite pattern or couldn’t be reproduced as it did not fit the inclusion criteria (i.e. measured in > 25 individuals). *For example,* VGF was decreased in AD in the EMIF-AD cohort while it was increased in ADNI. In addition, 14–3-3 proteins such as YWHAZ were increased in EMIF-AD, but none of these proteins were measured in ADNI. Therefore, we repeated the analysis in the 48 CSF panel [11] (supplementary Table 2). We found that the 10 synaptic proteins, including VGF and YWHAZ, measured in this panel now showed consisted findings with EMIF-AD MBD. Relative to controls, individuals with preclinical AD and prodromal AD showed lower levels of VGF, NPTX2 and SCG2 which was in concordance with the protein levels observed in EMIF-AD. In addition, YWHAZ was increased in prodromal AD compared to controls and preclinical AD which was also in agreement to the YWHAZ levels observed in EMIF-AD.

### Associations of synaptic protein levels with memory scores

**Fig. 3:**
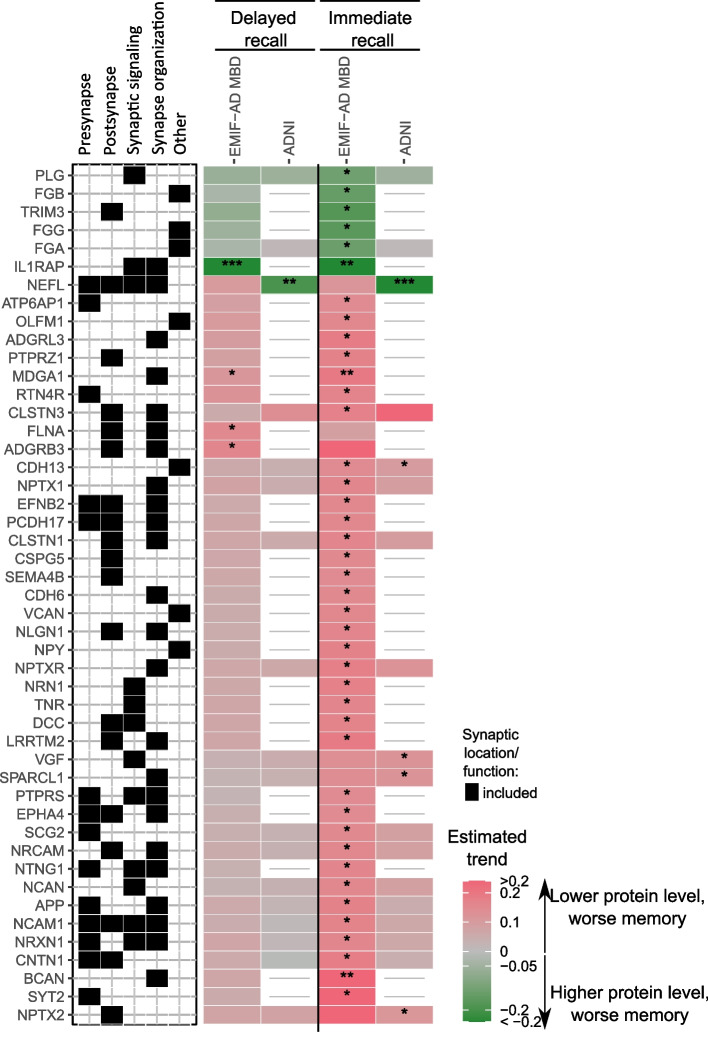
Synaptic proteins associated with memory independent of diagnostic group. Top proteins associated with memory across all diagnostic groups are shown. Associations were considered significant when proteins did not show an interaction with diagnostic group (interaction *p*-value ≥ 0.1) and had an association with memory function (*, *p*-value < 0.05; **, *p*-value < 0.01), ***, *p*-value < 0.001). Associations of all analysed synaptic proteins are provided in Supplementary Table 1

Here, we will summarise the results separately for immediate and delayed recall on memory tests, and separately report associations across diagnostic groups vs. associations that depended on diagnostic group (for full results, see Supplementary Table 1, and kwesenhagen.shinyapps.io/Synaptic_protein_associations_with_memory). First, a lower score on immediate recall was associated with lower concentrations of synaptic proteins in EMIF-AD (49 proteins) and ADNI (6 proteins) (Fig. 3). Of these associations, 1 protein (CDH13) between cohorts. A lower memory score was associated with higher levels of coagulation related proteins in EMIF-AD and with NEFL in ADNI.

**Fig. 4:**
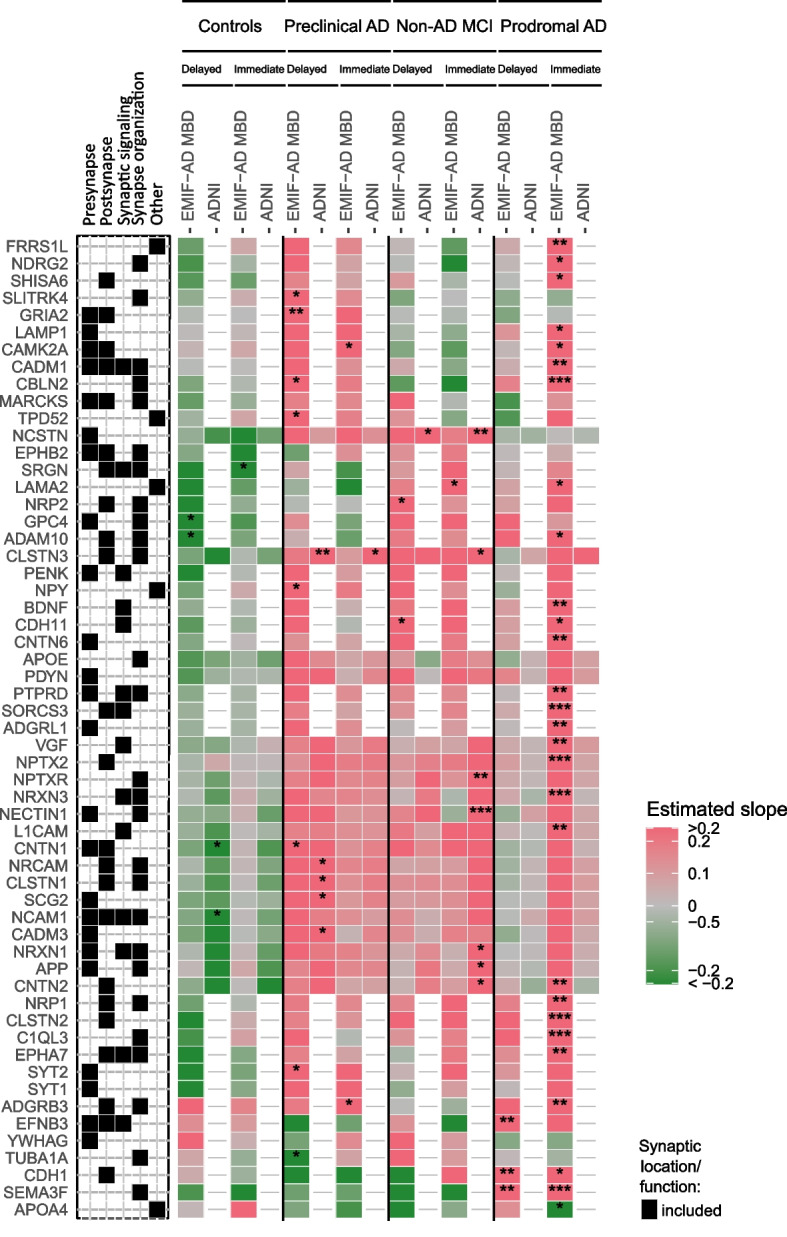
Memory-associated synaptic proteins stratified for amyloid and cognitive status. Associations between synaptic protein levels and delayed and immediate recall on word learning tests are shown stratified for diagnostic groups based on amyloid and cognitive status in EMIF-AD MBD and ADNI. Proteins which were related to memory function depending on diagnostic group in at least one cohort are shown. Associations were considered significant when proteins showed an interaction with diagnostic group on memory scores (*p*-value < 0.1) and showed an effect on memory function in diagnostic group-stratified analyses (*p*-value < 0.05). *, **, ***: significant effect of protein level on memory function in diagnostic group-stratified analyses (*, *p*-value < 0.05, **, *p*-value < 0.01; ***, *p*-value < 0.001). Synaptic category ‘Other’ refers to other synaptic locations and functions as detailed in Fig. 1c. Associations of all analysed synaptic proteins are provided in Supplementary Table 1

In EMIF-AD MBD, 30 synaptic proteins showed an interaction with diagnostic group on immediate recall scores. In prodromal AD, lower levels of 27 proteins were associated with lower immediate recall scores (Fig. 4), while higher levels of only (APOA4) was associated with worse immediate recall. Only 1 to 2 associations were found with immediate memory in controls, preclinical AD and non-AD MCI (Fig. 4, Supplementary Table 1).

In ADNI, 8 proteins showed an interaction with diagnostic group on immediate recall. Lower levels of 7 of these proteins (NECTIN, NCSTN, NCSTN, NPTXR, CNTN2, NRXN1, APP) were associated with worse immediate recall in non-AD MCI (NECTIN1, NCSTN, NCSTN, NPTXR, NRXN1 and APP; Fig. 4).

For delayed recall, lower levels of 3 proteins in EMIF-AD MBD and higher levels of 1 protein in ADNI were associated with worse delayed recall independent of diagnostic group (Fig. 3, Supplementary Table 1). For 22 proteins in EMIF-AD MBD, the association between synapse protein level and delayed recall differed between diagnostic groups. In controls, higher levels of GPC4 and ADAM10 were associated with lower recall scores. In preclinical AD, lower levels of 7 proteins were associated with lower delayed recall scores. In prodromal AD, lower levels of 3 proteins were associated with lower delayed recall scores (supplementary Table 1).

For 11 proteins in ADNI, the association between synapse protein level and delayed recall differed between diagnostic groups. In preclinical AD, lower levels of CLSTN1, CLSTN3, SCG2, CADM3 and NRCAM were associated with lower delayed recall scores (Fig. 4). In non-AD MCI and prodromal AD, lower levels of 2 to 3 proteins were associated with worse memory (Fig. 4, Supplementary Fig. 2). Supplementary Table 3 summarizes the findings of protein levels in prodromal AD and non-AD MCI and their associations with memory in both cohorts.

**Fig. 5:**
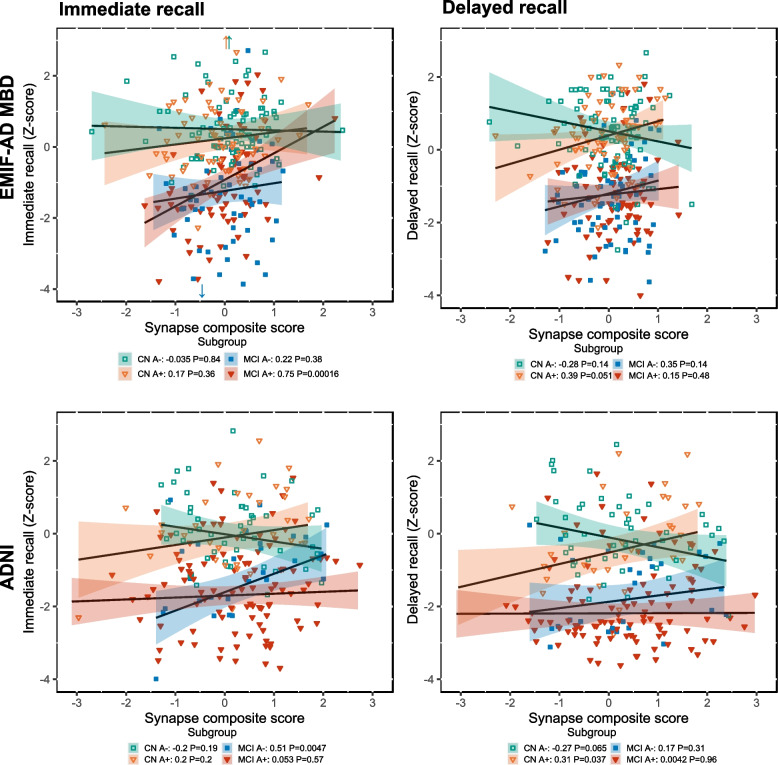
Associations of synaptic composite scores with immediate and delayed memory scores. Synaptic composites contain proteins associated with delayed recall (left) or immediate recall (right). CN A-, controls; CN A + , preclinical AD, MCI A-, non-AD MCI, MCI A + , prodromal AD

We finally performed enrichment analyses on proteins of which the association with memory scores differed between diagnostic groups. Synaptic proteins that were associated with immediate recall in non-AD MCI showed enrichment for synapse organization, and proteins related with immediate recall in prodromal AD showed enrichment for synapse organization and trans-synaptic signalling (Supplementary Fig. 2). Synaptic proteins related with delayed recall in preclinical AD showed enrichment for synapse organization and presynaptic functions (Supplementary Fig. 3). Figure 5 summarizes the associations of synaptic proteins with memory scores through composite scores of synaptic proteins associated with delayed and immediate recall in EMIF-AD MBD and ADNI.

## Discussion

Our main finding is that lower synaptic protein levels in CSF were associated with more impaired memory function in predementia AD. Therefore, treatment targeting synapses may be beneficial in early phases of AD.

### Reduced synaptic protein levels associated with worse memory functioning in preclinical AD

We found that the concentrations of five synaptic proteins (VGF, CDH2, NPTX2, BDNF and FXYD6) were reduced in preclinical AD relative to controls in EMIF-AD MBD. Reduced synaptic protein levels in CSF have been reported before in preclinical AD [24]. We extended previous studies by showing that lower delayed recall scores were associated with decreased synaptic protein levels in CSF in preclinical AD. These associations may reflect synapse loss [27, 28, 35–40]. The deterioration of synapses could in turn result in memory impairment [2, 8, 47]. Alternatively, this association can be bidirectionally interpreted, with higher levels of these proteins correlating with improved delayed recall scores. As the associated proteins did not show actual decreased levels compared to controls, it remains a possibility that higher protein levels could compensate for neuronal loss.

### Associations of synaptic proteins with immediate and delayed recall in prodromal AD

We found higher levels of NRGN in prodromal AD relative to controls which is in line with previous studies that showed that higher NRGN levels are associated with advanced disease stage and worse cognition [6, 19, 21]. In our study, however, there was only a slight trend towards a relationship between increased NRGN levels and memory function, which could imply that NRGN also relates to other aspects such as disease severity. Previous studies have shown an association of lower levels of in NPTX2 and VGF with worse cognition in non-demented individuals with AD and conversion to dementia [20, 22, 23]. This is in line with our results as we have found that decreases in NPTX2 and VGF levels were also related to worse cognition. These results suggests that these proteins may play a role in synaptic disturbance in AD which leads to cognitive impairment and reinforces the prognostic value of these proteins. Overall, the associations of synaptic proteins with immediate and delayed recall were similar, but substantially more associations with immediate recall than delayed recall were found in prodromal AD subjects suggesting that immediate recall may be more sensitive in early AD.

### Associations of synaptic proteins with immediate and delayed recall in non-AD MCI

In non-AD MCI, 11 proteins were associated with memory. The majority of memory-associated proteins in non-AD MCI (7 proteins) were specifically related to memory in non-AD MCI. Therefore, these may reflect synaptic dysfunction and memory decline caused by different disorders. For example, for diseases such as Parkinson’s disease (PD) and dementia with Lewy bodies (DLB) that can underly non-AD MCI [7, 26], aggregated alpha synuclein could induce synaptic disturbances [10, 46].

### Associations of synaptic proteins with delayed recall in controls

Rather surprisingly, in controls a higher concentration of 5 synaptic proteins were associated with worse delayed recall (Fig. 4). These proteins were not associated with memory in any of the other diagnostic groups. Some controls in EMIF-AD MBD had increased tau levels, which may have contributed to low memory scores in these individuals, as tau can impair synaptic plasticity and cause synaptic damage (Hu et al., n.d.; [17, 30, 33]). However, the proteins that did associate with memory in the other diagnostic groups showed associations of lower levels with lower memory scores. This suggests that the synaptic processes leading to impaired memory may differ in controls, non-AD MCI and Alzheimer’s disease. Moreover, we previously showed in cognitively normal individuals with normal AD biomarkers that those with higher synaptic proteins levels had a higher risk for AD pathology at follow-up such that the increase may indicate early AD [43].

### Difference in associations between ADNI and EMIF-AD MBD

In general, CSF proteins showed similar associations with memory scores when comparing the EMIF-AD MBD and ADNI datasets. An exception was prodromal AD, in which individuals from the EMIF-AD MBD cohort showed many associations of lower synaptic protein levels and lower immediate recall, which were not observed in ADNI. Potentially, the lack of reproducibility between the cohorts is due to the different word learning tests used in EMIF-AD MBD and ADNI. Prodromal AD individuals in ADNI also had on average lower delayed and immediate recall scores compared to EMIF-AD MBD (Table 1), likely because in ADNI impairment on a memory test was an inclusion criterion for the prodromal AD group, which was not the case in EMIF-AD MBD. The difference in disease severity in prodromal AD and the number and difference in measurement of proteins between both cohorts may also explain the lack of reproducibility. The latter is reinforced by the observation that the same proteins in the ADNI Caprion/RBM dataset showed a different association with cognitive function compared to the same proteins measured in the CSF 48 panel in ADNI.

### Memory associations of synaptic subcomponents and functions

In general, we observed that synaptic components (the pre- and post-synapse) and functions (synaptic signaling) showed similar memory associations. This suggests that generalized synapse loss underlies the associations of the observed synaptic proteins with memory. However, in preclinical AD, we observed enrichment of synapse organization among proteins that were associated with delayed recall in both cohorts. This could imply synapse (re-)organization might be a process involved in memory functioning in very early AD.

### Strengths and limitations

A limitation of this study is that cohorts showed different demographics (i.e. ADNI had an older population and lower memory scores), used different memory tests, procedures to normalize memory scores, different criteria for the diagnostic groups, and different proteomic platforms. Nonetheless, we found similar associations of synaptic protein levels with delayed recall in both cohorts and the validation CSF panel, which indicated these results are robust for cohort-dependent effects. For associations of the proteins with immediate recall in prodromal AD and non-AD MCI, we observed more heterogeneity between cohorts, perhaps reflecting that individuals with MCI form a heterogenous group. This stresses the importance of new, larger studies in individuals with prodromal AD.

Our data included only a part of the synaptic proteome (16.5%), so future studies should target a larger part of the synaptic proteome to investigate the role of synapses in memory functioning in more detail. Furthermore, while CSF proteomics allow simultaneous measurement of many proteins, it is not possible to determine whether alterations in concentrations are specific to particular anatomical brain structures. As such, future studies combining CSF proteomics and synapse PET would provide great anatomical detail of alterations in synaptic density. Lastly, the associations with memory performance we reported were not corrected for multiple testing. Instead, we tested associations with memory performance in two independent datasets which improves the robustness of the results. Strengths of our study are a the relatively large sample size from 2 independent studies and our analysis of synaptic changes in the very early stage of the disease that has received relatively little attention. Another strength is the publication of the memory associations of the analyzed synaptic proteins in an interactive online database (available at kwesenhagen.shinyapps.io/Synaptic_protein_associations_with_memory).

## Conclusions

CSF levels of synaptic proteins are decreased in early AD and were associated with memory loss. This indicates that the synapse may be an attractive target for therapeutic modulation in early AD. Further studies should therefore aim to study longitudinal relationships across different stages of AD.

## Supplementary Information

Supplementary Material 1

Supplementary Material 2

Supplementary Material 3

Supplementary Material 4

Supplementary Material 5

Supplementary Material 6

Supplementary Material 7

## Data Availability

EMIF-AD MBD data can be requested by contacting P.J. Visser and Stephanie Vos. ADNI data is publicly available and can be accessed via adni.loni.usc.edu.  ADNI data can be downloaded from adni.loni.usc.edu. The raw proteomic data from EMIF-AD MBD has been submitted to the ProteomeXchange Consortium through the PRIDE partner repository, under the dataset identifier 10.6019/PXD019910. Requests for access to other EMIF-AD MBD data should be directed to the authors. Data sharing restrictions may apply due to consent agreements from participants in each cohort and European GDPR regulations, which limit data sharing with several non-European countries. Statistical data are provided in the supplementary information files.

## Abbreviations

AD: Alzheimer’s disease
ADAM10: ADAM 10
ADGRB3: adhesion G protein-coupled receptor B3
ADNI: Alzheimer’s Disease Neuroimaging Initiative
AKR1A1: Aldo-keto reductase family 1 member A1
ANOVA: Analysis of variance
*APOE*: Apolipoprotein E
APP: amyloid precursor protein
AVLT: auditory verbal learning test
BDNF: brain-derived neurotrophic factor
CADM3: cell adhesion molecule 3
CDH1: cadherin-1
CDH2: cadherin-2
CDH11: cadherin-11
CDH13: cadherin-13
CDR: Clinical Dementia Rating
CERAD: Consortium to Establish a Registry for Alzheimer’s Disease wordlist
CLSTN1: calsyntenin-1
CLSTN3: calsyntenin-3
CN: cognitively normal
CNTN1: contactin-1
CNTN2: contactin-2
CSF: cerebrospinal fluid
DLB: dementia with Lewy bodies
EFNB3: ephrin-B3
EMIF-AD MBD: European Medical Information Network Alzheimer’s disease multi-modal biomarker discovery study
FCSRT: Free and Cued Selective Reminding Test
FDR: false discovery rate
FGA: fibrinogen alpha chain
FGB: fibrinogen beta chain
FGG: fibrinogen gamma chain
FLNA: filamin-A
FXYD6: FXYD domain containing ion transport regulator 6
GAP43: neuromodulin (also known as growth-associated protein 43)
GPC4: glypican-4
GRIA4: glutamate receptor 4
IL1RAP: IL-1 receptor accessory protein
LAMA2: laminin subunit alpha-2
LC/MS-MRM: liquid chromatography-mass spectrometry with multiple reaction monitoring
LRRTM2: leucine-rich repeat transmembrane neuronal protein 2
MCI: mild cognitive impairment
MDGA1: MAM domain-containing protein 3
MMSE: Mini-Mental State Examination
MRI: magnetic resonance imaging
NCSTN: nicastrin
NECTIN1: nectin-1
NEFL: neurofilament light
NLGN2: neuroligin-2
non-AD MCI: MCI with normal amyloid
NPTX2: neuronal pentraxin-2
NPTXR: neuronal pentraxin receptor
NRCAM: neuronal cell adhesion molecule
NRGN: neurogranin
NRXN1: neurexin-1
NRXN3: neurexin-3
PD: Parkinson’s disease
PET: positron emission tomography
PLG: plasminogen
RAVLT: Rey auditory verbal learning test
RBM: Rules-Based Medicine
SCG2: secretogranin-2
SEMA3F: Semaphorin-3F
SNAP25: synaptosomal-associated protein 25
SPARCL1: SPARC-like protein 1
SynGO: Synaptic Gene Ontologies
TMT: tandem mass tag
TPD52: tumor protein D52
TRIM3: tripartite motif-containing protein 3
VAPA: VAMP-associated protein A
VGF: neurosecretory protein VGF
YWHAE: 14-3-3 epsilon
YWHAH: 14-3-3 eta
YWHAZ: 14-3-3 zeta
