# Supplementary material for: Synaptic protein CSF levels relate to memory scores in individuals without dementia
Source: Alzheimers Res Ther. 2025 Mar 3;17:56. doi: 10.1186/s13195-025-01703-z (PMC11877693; doi:10.1186/s13195-025-01703-z)
Supplement: Supplementary file 4 — Supplementary Material 4 [file 13195_2025_1703_MOESM4_ESM.docx]

| **Supplementary Table 4.**  Ethical approval committee of each center in EMIF-AD MBD and ADNI. | | |
| --- | --- | --- |
| **Center** | **Cohort** | **Approval Committee** |
| Aristotle University, Thessaloniki | EMIF-AD MBD | Aristotle University of Thessaloniki Medical School Ethics Committee |
| Central Institute for Mental Health, Mannheim | EMIF-AD MBD | Ethics Committee of the Medical Faculty Mannheim, University of Heidelberg |
| GAP, San Sebastian | EMIF-AD MBD | Ethic and Clinical Research Committee Donostia |
| Hôpital Timone Adultes, Marseille | EMIF-AD MBD | Ethics committee Inserm and Aix Marseille University |
| Hospital Clínic de Barcelona IDIBAPS | EMIF-AD MBD | The Healthcare Ethics Committee of the Hospital Clínic |
| Hospital de la Santa Creu i Sant Pau, Barcelona | EMIF-AD MBD | Central Clinical Research and Clinical Trials Unit (UICEC Sant Pau) |
| INSERM, Toulouse | EMIF-AD MBD | INSERM Ethical Committee |
| IRCCS-FBF, Brescia | EMIF-AD MBD | Ethic Committee of the IRCCS San Giovanni di Dio FBF |
| IRCCS-SDN, Napels | EMIF-AD MBD | Comitato Etico IRCCS Pascale - Napoli |
| Karolinska Institutet, Stockholm | EMIF-AD MBD | Ethics Committee at Karolinska Institutet |
| Katholieke Universiteit, Leuven | EMIF-AD MBD | Ethische commissie onderzoek UZ/KU Leuven |
| Lausanne University Hospital, Lausanne | EMIF-AD MBD | Research Ethics Committee Lausanne University Hospital |
| Maastricht University, Maastricht | EMIF-AD MBD | Medical ethical committee Maastricht University Medical Center |
| Rigshospitalet, Copenhagen | EMIF-AD MBD | Committee on Health Research Ethics, Region of Denmark |
| University of Mediterranean, Marseille | EMIF-AD MBD | Ethics committee of Mediterranean University |
| University of Lille, Lille | EMIF-AD MBD | University of Lille Ethics committee |
| University of Leipzig, Leipzig | EMIF-AD MBD | Ethical Committee at the Medical Faculty, Leipzig University |
| University of Essen, Essen | EMIF-AD MBD | Ethical Committee at the Medical Faculty, University Hospital Essen |
| University of Antwerp, Antwerp | EMIF-AD MBD | Ethics committee University of Antwerp |
| University of Genoa, Genoa | EMIF-AD MBD | Ethical Committee of University of Genoa |
| University of Gothenburg, Gothenburg | EMIF-AD MBD | Ethics Committee, University of Gothenburg |
| University of Perugia, Perugia | EMIF-AD MBD | Human ethics Committee of the University of Perugia |
| VU Medical Center, Amsterdam | EMIF-AD MBD | Medical ethics committee VU Medical Center |
| Albany Medical Center, Albany | ADNI | Albany Medical Center Committee on Research Involving Human Subjects Institutional Review Board |
| Boston University Medical Center, Boston | ADNI | Boston University Medical Campus and Boston Medical Center Institutional Review Board |
| Butler Hospital, Providence | ADNI | Butler Hospital Institutional Review Board |
| Cleveland Clinic, Cleveland | ADNI | Cleveland Clinic Institutional Review Board |
| Columbia University Medical Center, New York | ADNI | Columbia University Medical Center Institutional Review Board |
| Duke University Health System, North Carolina | ADNI | Duke University Health System Institutional Review Board |
| Emory University, Atlanta | ADNI | Emory Institutional Review Board |
| Georgetown University, Washington D.C. | ADNI | Georgetown University Institutional Review Board |
| Houston Methodist, Housten | ADNI | Houston Methodist Institutional Review Board |
| Howard University, Washington D.C. | ADNI | Howard University Office of Regulatory Research Compliance |
| Icahn School of Medicine, Mount Sinai | ADNI | Program for the Protection of Human Subjects |
| Indiana University, Bloomington | ADNI | Indiana University Institutional Review Board |
| Baylor College of Medicine, Houston | ADNI | Institutional Review Board of Baylor College of Medicine |
| Jewish General Hospital, Quebec | ADNI | Jewish General Hospital Research Ethics Board |
| Johns Hopkins Medicine, Baltimore | ADNI | Johns Hopkins Medicine Institutional Review Board |
| Rhode Island Hospital, Providence | ADNI | Lifespan - Rhode Island Hospital Institutional Review Board |
| Mayo Clinic, Rochester | ADNI | Mayo Clinic Institutional Review Board |
| Mount Sinai Medical Center, Mount Sinai | ADNI | Mount Sinai Medical Center Institutional Review Board |
| Nathan Kline Institute for Psychiatric Research & Rockland Psychiatric Center, Orangeburg | ADNI | Nathan Kline Institute for Psychiatric Research & Rockland Psychiatric Center Institutional Review Board |
| New York University Langone Medical Center, New York | ADNI | New York University Langone Medical Center School of Medicine Institutional Review Board |
| Northwestern University, Evanston | ADNI | Northwestern University Institutional Review Board |
| Oregon Health and Science University, Oregon | ADNI | Oregon Health and Science University Institutional Review Board |
| Mass General Brigham, Boston | ADNI | Partners Human Research Committee Research Ethics |
| Sunnybrook Hospital, Toronto | ADNI | Board Sunnybrook Health Sciences Centre |
| Saint Francis University, Loretto | ADNI | Roper St. Francis Healthcare Institutional Review Board |
| Rush University Medical Center, Chicago | ADNI | Rush University Medical Center Institutional Review Board |
| St. Joseph’s Medical Center, Phoenix | ADNI | St. Joseph's Phoenix Institutional Review Board |
| Stanford University, Standford | ADNI | Stanford Institutional Review Board |
| The Ohio State University, Columbus | ADNI | The Ohio State University Institutional Review Board |
| University Hospitals Cleveland Medical Center, Cleveland | ADNI | University Hospitals Cleveland Medical Center Institutional Review Board |
| University of Alabama, Tuscaloosa | ADNI | University of Alabama Office of the IRB |
| University of British Columbia, Vancouver | ADNI | University of British Columbia Research Ethics Board |
| University of California Davis, Davis | ADNI | University of California Davis Institutional Review Board Administration |
| University of California Los Angeles, Los Angeles | ADNI | University of California Los Angeles Office of the Human Research Protection Program |
| University of California San Diego, San Diego | ADNI | University of California San Diego Human Research Protections Program |
| University of California San Francisco, San Francisco | ADNI | University of California San Francisco Human Research Protection Program |
| University of Iowa, Iowa City | ADNI | University of Iowa Institutional Review Board |
| University of Kansas Medical Center, Kansas City | ADNI | University of Kansas Medical Center Human Subjects Committee |
| University of Kentucky, Lexington | ADNI | University of Kentucky Medical Institutional Review Board |
| University of Michigan, Ann Arbor | ADNI | University of Michigan Medical School Institutional Review Board |
| University of Pennsylvania, Philadelphia | ADNI | University of Pennsylvania Institutional Review Board |
| University of Pittsburgh, Pittsburgh | ADNI | University of Pittsburgh Institutional Review Board |
| University of Rochester, Rochester | ADNI | University of Rochester Research Subjects Review Board |
| University of South Florida, Tampa | ADNI | University of South Florida Institutional Review Board |
| University of Southern California, Los Angeles | ADNI | University of Southern California Institutional Review Board |
| UT Southwestern Medical Center, Dallas | ADNI | UT Southwestern Institutional Review Board |
| VA Long Beach Healthcare System, Long Beach | ADNI | VA Long Beach Healthcare System Institutional Review Board |
| Vanderbilt University Medical Center, Nashville | ADNI | Vanderbilt University Medical Center Institutional Review Board |
| Wake Forest School of Medicine, Winston-Salem | ADNI | Wake Forest School of Medicine Institutional Review Board |
| Washington University, St. Louis | ADNI | Washington University School of Medicine Institutional Review Board |
| Wayne State University, Detroit | ADNI | Western Institutional Review Board |
| Western University, London (Canada) | ADNI | Western University Health Sciences Research Ethics Board |
| Yale University, New Haven | ADNI | Yale University Institutional Review Board |
