# Supplementary material for: Synaptic protein CSF levels relate to memory scores in individuals without dementia
Source: Alzheimers Res Ther. 2025 Mar 3;17:56. doi: 10.1186/s13195-025-01703-z (PMC11877693; doi:10.1186/s13195-025-01703-z)
Supplement: Supplementary file 5 — Supplementary Material 5 [file 13195_2025_1703_MOESM5_ESM.docx]

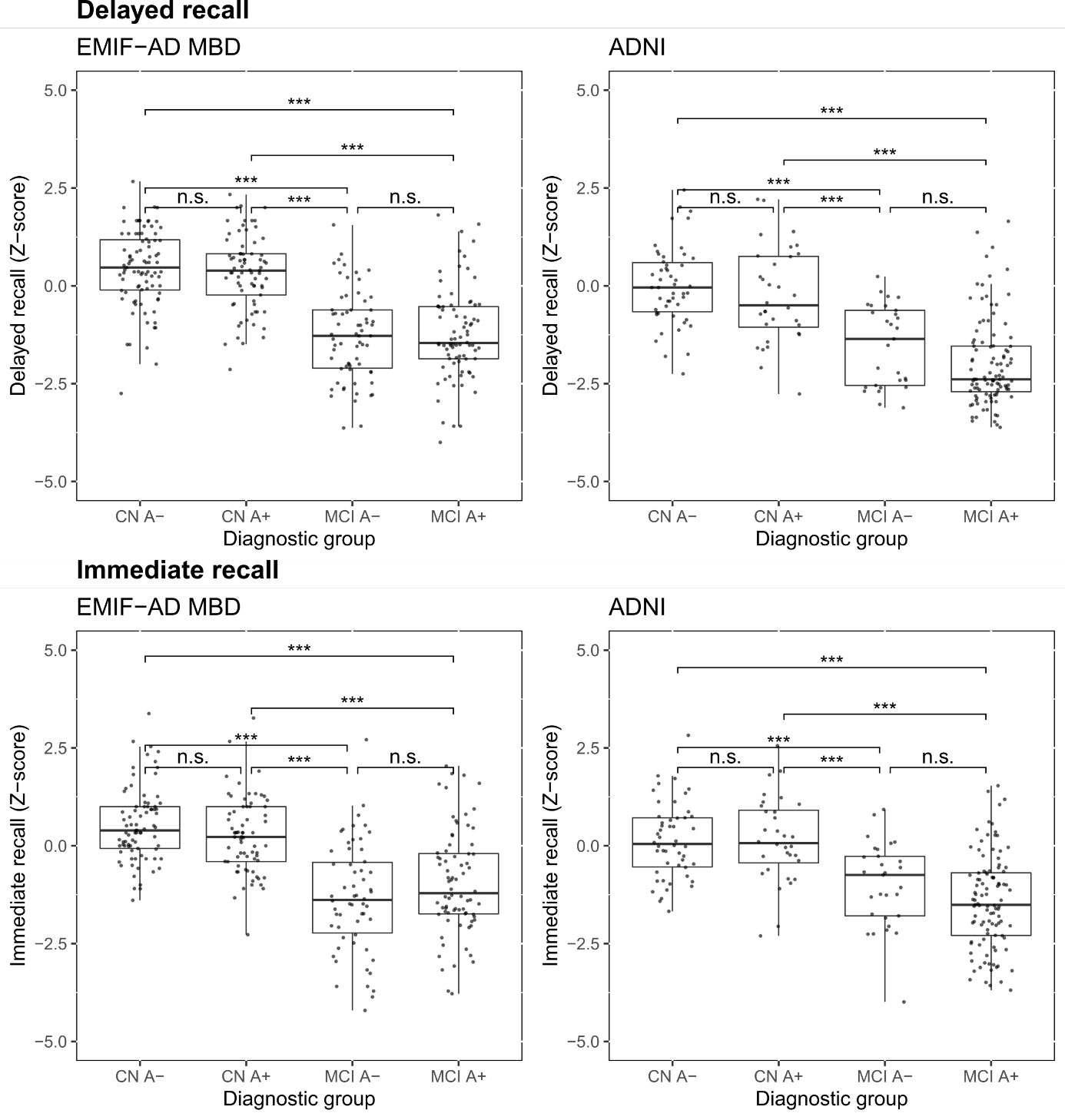


**Supplementary Figure 1: Immediate and delayed recall scores in EMIF-AD MBD and ADNI.** Immediate and delayed recall scores on word learning tests are Z-scores adjusted for age, sex and education (further details provided in the Methods). The box of the boxplot indicates 25^th^ percentile, median and 75^th^ percentile, whiskers indicate 1.5x interquartile range. N.s., not significant; *, P-value < 0.05; **, P-value < 0.01; ***, P-value < 0.001. CN A-, controls; CN A+, preclinical AD, MCI A-, Non-AD MCI, MCI A+, prodromal AD.
