# Supplementary material for: Synaptic protein CSF levels relate to memory scores in individuals without dementia
Source: Alzheimers Res Ther. 2025 Mar 3;17:56. doi: 10.1186/s13195-025-01703-z (PMC11877693; doi:10.1186/s13195-025-01703-z)
Supplement: Supplementary file 6 — Supplementary Material 6 [file 13195_2025_1703_MOESM6_ESM.docx]

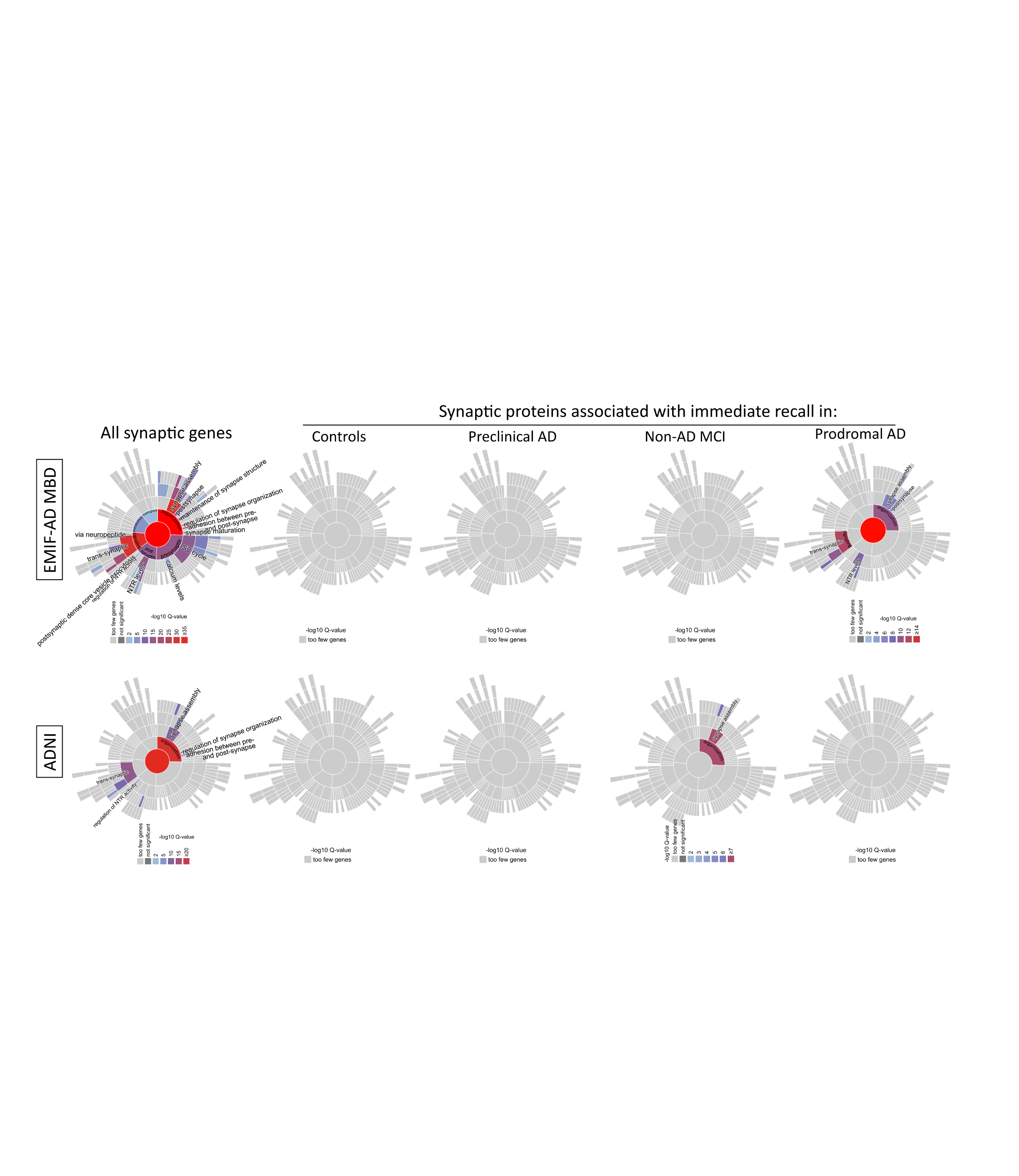


**Supplementary Figure 2: Relation of immediate recall-associated proteins with synaptic processes.** Enrichment analysis of synaptic processes, tested using the SynGO website (see Methods for further details). The enrichment analyses were conducted separately in each diagnostic group, selecting proteins of which the interaction between protein level and diagnostic group had a p-value < 0.1 and which additionally were related to immediate recall in that diagnostic group (p-value < 0.05). Enrichment analysis of all synaptic genes is shown for an overview of synaptic proteins included in each dataset.
